# Phytotoxicity of Graphene Family Nanomaterials and Its Mechanisms: A Review

**DOI:** 10.3389/fchem.2019.00292

**Published:** 2019-05-01

**Authors:** Qinghai Wang, Cui Li, Yu Wang, Xiaoe Que

**Affiliations:** ^1^Beijing Research and Development Center for Grass and Environment, Beijing Academy of Agriculture and Forestry Sciences, Beijing, China; ^2^State Key Laboratory of Multiphase Complex Systems, Institute of Process Engineering, Chinese Academy of Sciences, Beijing, China; ^3^Institute of Desertification Studies, Chinese Academy of Forestry, Beijing, China

**Keywords:** nanomaterials, plant, phytotoxicity, influencing factor, toxicity mechanisms

## Abstract

Graphene family nanomaterials (GFNs) have experienced significant development in recent years and have been used in many fields. Despite the benefits, they bring to society and the economy, their potential for posing environmental and health risks should also be considered. The increasing release of GFNs into the ecosystem is one of the key environmental problems that humanity is facing. Although most of these nanoparticles are present at low concentrations, many of them raise considerable toxicological concerns, particularly regarding their accumulation in plants and the consequent toxicity introduced at the bottom of the food chain. Here, we review the recent progress in the study of toxicity caused by GFNs to plants, as well as its influencing factors. The phytotoxicity of GFNs is mainly manifested as a delay in seed germination and a severe loss of morphology of the plant seedling. The potential mechanisms of phytotoxicity were summarized. Key mechanisms include physical effects (shading effect, mechanical injury, and physical blockage) and physiological and biochemical effects (enhancement of reactive oxygen species (ROS), generation and inhibition of antioxidant enzyme activities, metabolic disturbances, and inhibition of photosynthesis by reducing the biosynthesis of chlorophyll). In the future, it is necessary to establish a widely accepted phytotoxicity evaluation system for safe manufacture and use of GFNs.

## Introduction

Graphene family nanomaterials (GFNs), a typical representative of two-dimensional carbon nanomaterials (CNMs), have been widely used in various fields, including energy storage, nanoelectronic devices and batteries, biomedical applications, biosensors, cell imaging, drug delivery, and tissue engineering (Ou et al., 2016). GFNs include few-layer-graphene (FLG), ultrathin graphite, graphene oxide (GO), reduced graphene oxide (rGO), and graphene nanosheets (GNS) (Sanchez et al., 2012). Furthermore, GFNs can serve as an important building platform for constructing various supramolecular products that have several advantageous applications (Dreyer et al., 2010; Zhou et al., 2013). However, these carbon nanomaterials will inevitably be released into the environment during their production, transport, consumption, and disposal. Their environmental use for wastewater and drinking water treatment will likely lead to considerable release of the aforementioned materials (Zhao et al., 2014). There has been considerable research regarding the phytotoxicity of GFNs, but far less research on the realistic release amount and concentration in the environmental media (air, water, and soil). Yan et al. (2019) reported that the maximum release amount of graphene was 1.6 mg/kg from graphene-polyethylene composite films applied in food packaging, confirming the release of GFNs. Miralles et al. (2012) summarized the release pathways of engineered nanomaterials into the natural environment as follows: their use in environment remediation, as delivery systems in agriculture, as biosensors, and as release from medical and cosmetic applications; as well as accidental release (e.g., atmospheric emissions, leaching from sewage sludge, etc.). This information is helpful in understanding the release pathways of GFNs. Early in 2005, researchers conducted an evaluation of nanomaterials regarding human health risks (Thomas and Sayre, 2005). Hereafter, considering its persistent and hydrophobic properties, and dramatically increasing production, (Arvidsson et al., 2013) proposed that the fate of graphene in the environment and its toxicity should be further studied. Many researchers have so far expressed concern about the potential human health and ecological risks resulting from the manufacture and use of GFNs (Gilbert, 2009; Suárez-Iglesias et al., 2017; Chen et al., 2018b; Naasz et al., 2018). Currently, most research is focused on the effects of GFMs on humans, small mammals, invertebrates, and aquatic organisms, and little research has investigated their effects on plants (Lee et al., 2016). As primary producers, plants play a major role in the ecosystem. They not only interact directly with the soil, water, and atmospheric compartments of the environment (Miralles et al., 2012), but also provide food for people and other animals. It is also the starting point for the bioaccumulation of toxic substances. Therefore, it is likely that nanoparticles are gradually enriched to higher levels of the food chain, leading to toxic effects in organisms further up the chain (Yang and Zhao, 2013). Understanding the hazards of nanomaterials (e.g., toxicity, mutagenicity, impacts on ecosystem services), and the underlying toxicity mechanisms, is a basis for the more focused study of the processes required to control their exposure (Wiesner et al., 2009). Therefore, we should pay more attention to the phytotoxicity of GFNs and its influencing factors, as well as its potential toxicity mechanisms. The purpose of this article is to critically review the existing literature on the phytotoxicity, toxic influencing factors, and toxicological mechanisms of GFNs. Some reviews have been written on the toxic effects of GFNs in several organs and cell models (Ou et al., 2016). In addition, toxicity, uptake, and translocation of engineered nanomaterials in vascular plants (Miralles et al., 2012). We believe that a comprehensive review is necessary to recognize emerging trends and to discuss existing knowledge gaps on the toxicity of GFNs to plants, especially crop plants.

## Phytotoxicity of GFNs

**Figure 1:**
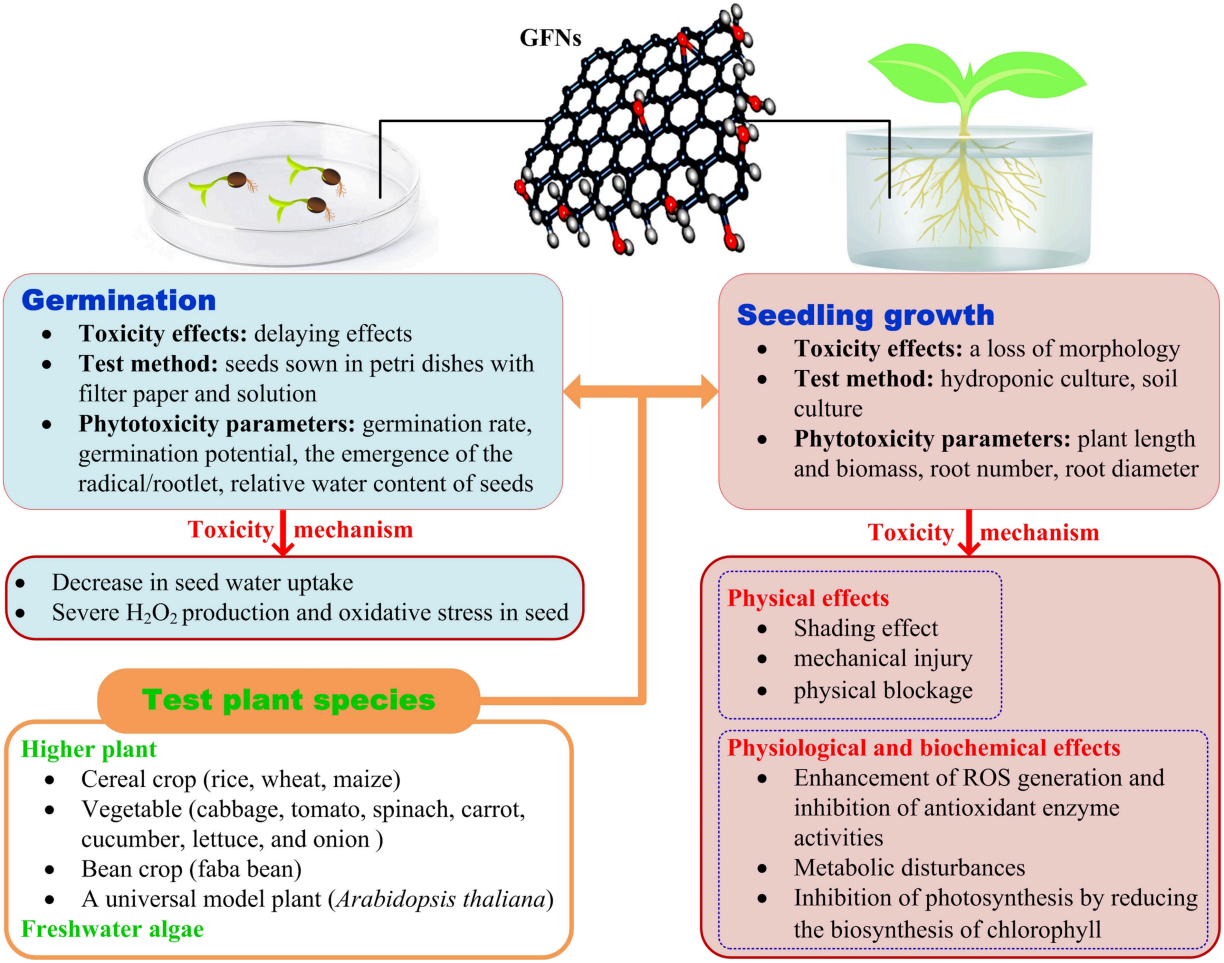
Schematic diagram of the phytotoxicity and possible mechanisms of GFNs.

Due to possible direct human exposure through the food chain, crop plants have been chosen as test subjects in most research on phytotoxicity of GFNs. The following review is mainly focused on the findings obtained regarding crop plants. The toxicity of GFNs to plants is summarized in Figure 1.

### GFNs Fate in Plants

Graphene can be transferred from wheat roots to shoots and enter the cytoplasm and chloroplasts (Hu et al., 2014c); however, GO accumulation was not observed in the root cells of wheat (Chen et al., 2018a). Furthermore, GO did not accumulate in the seedlings of spinach and chive from if their seeds were treated with 50 mg/L GO (He et al., 2018). In another study, Zhao et al. (2015) found that GO in the range of μg/L accumulated in root hair and root parenchyma cells; however, it did not translocate into the stem or leaves of *Arabidopsis thaliana*. This finding was supported by Chen et al. (2017); GO was readily absorbed by the plant roots, but the absorbed GO showed limited upward translocation. Different quantitative and distributional trends between the two graphene materials in pea plants were observed by Chen et al. (2019). Their study showed that rGO was translocated into leaves after being absorbed by the roots. The uptake amount in the root tended to stabilize at 15-day exposure, and the cumulative amount in the leaves was higher than that in the roots at 20-day exposure, reaching accumulation amounts up to mg level. Conversely, GO mainly accumulated in the roots and low levels in leaves. Besides root accumulation and translocation from root to shoot, Huang et al. (2018) further discovered that ~9% of the accumulated FLG was degraded to CO_2_ in the rice plant, and that the hydroxyl radical in the leaf played an important role in degrading FLG. Earlier studies have demonstrated that CO_2_ was the final product of the complete enzymatic catalyzed oxidation of GO (Kotchey et al., 2011), and H_2_O_2_ was a key component of this degradation process (Xing et al., 2014). In plants, H_2_O_2_ plays an important role in regulating biotic and abiotic stress responses (Sun et al., 2018). Thus, given the widespread presence of H_2_O_2_ in plants, they can potentially eliminate accumulated GFNs and could be used as phytoremediation agents for environmental clean-up.

### Toxic Effects on Plant Germination

Generally, GFNs produce a delaying effect in plant germination. For example, in rice seeds treated with 50 mg/L of graphene, germination started 3 days after the control group (Nair et al., 2012). A later study confirmed that the increase in graphene stress (≤ 200 mg/L) induced a delay in the initiation of the germination process in rice, but had no negative effects on the final germination percentage (Liu et al., 2015). A similar delay occurred in the appearance of the cotyledons and the root system of tomato, cabbage, and red spinach seeds treated with graphene (Begurn et al., 2011). However, tomato seeds exposed to graphene at concentrations as low as 40 mg/L obtained rapid seed germination and higher germination rates, which was attributed to the penetration of the seed coat by the graphene, thus facilitating water uptake (Zhang et al., 2015b). GO also significantly stimulated plant germination at 50 mg/L because its hydrophobic sp^2^ domains transported more water to the seed in the soil (He et al., 2018). GO at concentrations in the order of μg/L (10–1000 μg/L) had no obvious influence on the germination of *Arabidopsis* seeds (Zhao et al., 2015). When GO concentration was up to 100 mg/L, the rice germination percentage insignificantly decreased; the effect became significant at 500 mg/L GO (Liu et al., 2015). Similar to rice, the germination of wheat seeds was inhibited owing to GO concentrations exceeding 400 mg/L (Chen et al., 2017).

### Toxic Effects on Plant Growth

The common toxicity symptoms observed in plants exposed to GFNs are a severe loss of morphology and decreases in growth parameters, such as root and shoot length, root number, root diameter, and biomass production (Table 1). For instance, the morphology of rice seedlings was significantly inhibited if graphene concentration reached 100 mg/L (Liu et al., 2015). GO also adversely affected biomass accumulation and stem elongation in wheat seedlings (Chen et al., 2017). After exposure to 50 and 500 mg/kg rGO, negative effects on the shoot height and root length of rice seedlings were observed by Hao et al. (2018); in addition, the root diameter and the number of cells in the transverse section significantly decreased. However, GO in the range of μg/L did not cause significant changes in shoot and root development of *Arabidopsis* seedlings, or flowering time (Zhao et al., 2015). These apparently contrasting results suggest that the toxic effects of GFNs are associated with exposure concentrations.

**Table 1:** Toxic effects on the germination and growth of crop plants.

**Plant species**	**GFNs**	**Exposure concentration**	**Exposure time**	**Toxic symptoms of growth**	**References**
Rice (*Oryza sativa*)	Graphene	100 and 200 mg/L	16 days	Inhibition of root and stem length, adventitious root number, and root and stem fresh weight	Liu et al., 2015
	rGO	50, 500 mg/kg	30 days	Reduced shoot height and root length, decreased root diameter and number of cells in transverse section	Hao et al., 2018
wheat (*Triticum aestivum*)	GO	>1000 μg/mL	9 days	Decrease in root length, shoot length and relative biomass; obvious damage to plant tissue structures	Chen et al., 2018a
	Graphene	250, 500, 1000, and 1500 mg/L	30 days	Root hair reduction, oxidative burst, photosynthesis inhibition, and nutritional disorder	Zhang et al., 2016
		200 mg/L	5 days	Inhibition in the number of wheat roots	Hu et al., 2014c
Maize (*Zea mays*)	GO	100, 500, 1500 mg/L	15 days	Significant decrease of shoot and root weight	Yin et al., 2018
*Brassica napus*	GO	25–100 mg/L	15 days	Shorter seminal root length	Cheng et al., 2016
		50-100 mg/L		Decrease in fresh root weight	
Cabbage (*Brassica oleracea*), tomato (*Lycopersicon esculentum*), red spinach (*Amaranthus tricolor* and *A. lividus*)	Graphene	500–2000 mg/L	20 days	Significant inhibition of plant growth and biomass. Decrease in the number and size of leaves	Begurn et al., 2011

## Factor

The potential effects of GFNs depend on many factors, such as their physicochemical, the exposure concentration and time, and the plant species, which deserve further attention.

### Physicochemical Properties of GFNs

The biological impacts of nanomaterials are dependent on their size, chemical composition, surface structure, solubility, shape, and aggregation. Of these properties, size and surface area are important characteristics from a toxicological perspective; a small size and a large surface area increase the uptake and interaction with biological tissues, thus increasing the probability of generating adverse biological effects in living cells (Nel et al., 2006).

Because the large size of GO sheets hinders its translocation from the roots to the stem and leaves, GO bioaccumulation was much lower than that of fullerenol in wheat roots (Chen et al., 2017). Using freshwater algae as a test plant, Zhao et al. (2017) investigated the toxicity of GFNs based on their different physicochemical properties and colloidal behaviors. They found that GO with abundant functional groups could adsorb more macronutrients (N, P, Mg, and Ca) from the culture medium than rGO, thus leading to stronger nutrient depletion-induced indirect toxicity; rGO could directly penetrate into algal cells, but GO, with more flexible sheets, could not. In addition, GO had a significant shading effect on algal growth due to its good dispersibility and transformation. Compared to graphene and GO, hydrated graphene ribbon (HGR) not only promoted the germination rate of aged seed, but also increased root differentiation; the disordered layer structures of HGR played a key role in this process (Hu and Zhou, 2014). The observations of Chen et al. (2018a) showed that GO induced obvious toxic symptoms in wheat, while amine-functionalized GO was non-toxic and enhanced plant growth. They inferred that the introduction of amines could decrease the surface electrical resistivity of GO, creating higher electronic conductivity, and activating bioactivity in plant cells.

### Exposure Concentration and Time

The general effect of GFNs on plant growth is dose-dependent. Graphene at 5 mg/L promoted the number of adventitious roots, and increased the root and shoot fresh weight of rice seedlings; however, at a concentration of 50 mg/L, it significantly inhibited the stem length and fresh shoot weight (Liu et al., 2015). Graphene at 500 mg/L resulted in only a slight decrease in root and shoot length of tomato, cabbage, and red spinach, whereas a marked inhibition was induced by graphene at concentrations up to 2,000 mg/L (Begurn et al., 2011). After a 10-day exposure to GO, the lower concentrations (5 and 10 mg/L) had no significant effect on root length and fresh weight, but the higher concentrations (50 and 100 mg/l) showed inhibited root growth (Cheng et al., 2016). However, this effect is not completely concentration-dependent. Anjum et al. (2014) found that GO at 1600, 200, and 100 mg/L significantly inhibited the germination rate and root length of the faba bean (*Vicia faba*), while the health status of the plant was improved with exposure to GO at 400 and 800 mg/L. Their previous study assessed the tolerance of faba bean to GO, in which the plant showed a significantly higher sensitivity to GO at 1,600, 200, and 100 mg/L, and its tolerance increased when exposed to 400 and 800 mg/L concentrations (Anjum et al., 2013). Their further investigations indicated that the sensitivity and/or tolerance of the plant to GO depended on the cellular GSH redox system. Additionally, the concentration-dependent toxicity of GFNs is also related to exposure time. Zhang et al. (2016) found that long-term graphene exposure (30 d) caused wheat leaf deformities and yellowing, whereas no distinct alterations in leaf elongation were found after short-term exposures (24 or 48 h). This exposure time-dependent toxicity was also observed by Zhao et al. (2017). In their investigation, the growth inhibition of GFNs to freshwater algae highly increased with increasing exposure times (24–96 h).

### Plant Species

Heretofore, only a few studies have been designed to compare the toxicity of GFNs to different plants. The hydroponics experiments conducted by Begurn et al. (2011) indicated that graphene had little or no significant toxic effect on lettuce seedlings, but significantly inhibited the growth and biomass of tomato, cabbage, and red spinach seedlings under the same conditions. Among these selected vegetable species, tomato seedlings exhibited the highest sensitivity to graphene, according to the root and shoot weight data.

### Interaction With Co-existing Pollutants

Compared with studies on the ecotoxicity of single nanomaterials, relatively little research has focused on the interaction of nanoparticles and other contaminants (Zhang et al., 2018). In general, GFNs coexist with other pollutants in the natural environment. Heavy metals and organic pollutants (OPs) are common in the water and soil environment. The interactive toxic effects are different when GFNs are combined with these pollutants; these are termed additive or antagonistic effects. The research of Hu et al. (2014a) found that GO amplified the phytotoxicity of As in wheat. It further revealed the main mechanisms of indirect toxicity of GO: (a) enhancing the uptake of GO and As by damaging cellular structures and electrolyte leakage, and (b) promoting the transformation of As^5+^ to high-toxicity As^3+^. Conversely, another investigation found that GO alleviated the inhibitive effects of Cd^2+^ on the seminal root and bud growth of rice, which possibly resulted from Cd^2+^ adsorption in available contact sites or accumulation in the interlayer space of GO (Yin et al., 2018). Early researchers had already noticed the strong attractive forces between Cd^2+^ and GO, and had used GO as a sorbent for heavy metal removal from waste water (Tan et al., 2015). Lingamdinne et al. (2016) found that the adsorption occurred through physical and chemical interactions between heavy metal ions and oxygen-containing surface functional groups, and the π-π bond electrons of GO. Moreover, functionalized GO exhibited significantly higher adsorption capacity (Pirveysian and Ghiaci, 2018). However, once desorption occurs, high adsorption capacity implies the potential release, thus presenting a high risk to public health and the environment (Yang and Xing, 2007). GFNs have also been widely used to remove OPs from the environment (Chowdhury and Balasubramanian, 2014; Amaranatha et al., 2015; Zhang et al., 2015a). Although no direct data currently suggest that interaction of GFNs and OPs enhances phytotoxicity of either, it has been confirmed that GO can serve as an insecticide carrier to enhance contact toxicities (Wang et al., 2019). This synergistic mode of adsorption-delivery-release is most likely equally effective for plants. Moreover, previous research demonstrated that C_60_ fullerenes significantly increase the bioaccumulation of DDE (dichlorodiphenyldichloroethylene, DDT metabolite) into three selected food crops (De La Torre-Roche et al., 2012). These findings indicate that the carbon nanomaterials can affect the accumulation and bioavailability of co-existing pollutants, and thereby be regarded as a toxic alert to plants.

## Toxicity Mechanism of GFNs

The toxicity mechanism of GFNs to plants is summarized in Figure 1.

### Physical Effects

The main physical mechanisms for the phytotoxicity of GFNs include the shading effect, mechanical injury, and physical blockage. Both shading effect and mechanical injury were observed by Zhao et al. (2017). They found that the dispersed and darkened GO reduced light transmittance, thus decreasing the available light required to support plant growth, resulting in approximately 16% of growth inhibition; and more interestingly, direct penetration into algal cells by graphene materials was discovered for the first time. The physical blockage is closely related to the size of GFNs. If nanoparticle diameter is larger than the diameter of root cell wall pores, particles will accumulate at the root surfaces and form surface layers, thus decreasing the hydraulic conductivity and uptake of nutrients (Asli and Neumann, 2009).

### Physiological and Biochemical Effects

GFNs enhance the generation of reactive oxygen species (ROS) and inhibit antioxidant enzyme activities, resulting in oxidative stress, which has been recognized as one of the most important mechanisms in growth-limiting effects on plants (Hu et al., 2014b; Zhang et al., 2016; Chen et al., 2017). GO also caused metabolic disturbances linked to key biological processes, such as inhibiting carbohydrate and amino acid metabolisms, and increasing the ratios of unsaturated to saturated fatty acids, changing the flux of nitrogen metabolism (Hu et al., 2014b).

It is well-known that photosynthesis is critical for plant survival and growth. Graphene significantly inhibited the biosynthesis of chlorophyll and decreased chlorophyll content in plants, leading to impaired photosynthesis and reduced growth (Hu et al., 2014c; Zhang et al., 2016). Hu et al. (2014c) also found that glyconic acid and aconitic acid were upregulated by graphene, and these metabolites were negatively correlated with the biosynthesis of chlorophyll.

## Problems and Prospects

Plant bioassay, the physicochemical properties of GFNs, and toxicity endpoints, are key factors in toxicity evaluation. At present, most research has focused on crop species (Miralles et al., 2012), and competitive toxicity assays between GFNs has only been conducted for lower plant forms. More risk assessments in a large range of plants must be systematically investigated. In order to develop GFNs for further use in various fields, many efforts have already been initiated on functionalization of GFNs by supramolecular approaches (Chen et al., 2015; Gobbi et al., 2017); such modifications create multiple and complex properties in GFNs (Xu et al., 2018). However, little is known about the relevance of phytotoxicity with the properties of GFNs, and a fundamental understanding of this relationship is essential to their applications. Additionally, functionalized, and non-functionalized nanomaterials exhibited significantly different toxicity to several crop species, thereby requiring future study to evaluate the potential toxicity of both forms Cañas et al. (2008). Currently, indicators of plant germination, growth, and physiology have been often used to evaluate the toxicity of GFNs in most existing studies. These visually identifiable and practical indicators are easy to obtain, but might not fully reflect the toxic effects and mechanisms of GFNs; in addition, they have a different sensitivity to GFNs. For instance, the number of roots was more sensitive to graphene than seed germination or fresh weight (Hu et al., 2014c). Therefore, endpoint selection in toxicity tests is very important, and studies at the molecular level are needed to develop a deep understanding of the toxicity mechanisms of GFNs in plants.

Compared to assessing their toxicity, the uptake, transport, distribution, and degradation of GFNs within plants remains poorly understood (Huang et al., 2018). Their transformation pathways and fate in water/soil-plant systems requires additional research, which will contribute to prevention of environmental risks. It is possible that the phytotoxicity of GFNs could lead to crop yield reduction. However, the current research is mainly focused on responses of crop plants to GFNs at the seedling stage, there is no direct data to support this. Therefore, relevant researches should cover the whole of the growth period of each tested crop.

Taken together, although a large number of phytotoxicity assays for GFNs have been carried out, there is a big difference in terms of the selected plant species, the growth stages of the plants, the plant material culture methods (soil culture or hydroponics), and the exposure time between these toxicity tests; this leads to a lack of comparability of the assessment results. Moreover, the systematical assessment of GFNs phytotoxicity is hampered by this limited comparability. Therefore, it is necessary to establish a phytotoxicity evaluation system for GFNs, like the U.S. EPA or OECD guidelines for chemical testing.

Once GFNs are in use, release into the environment should be avoided to the largest extent possible by a rational scientific approach. For this purpose, the cooperation of chemists and biologists is crucial to implementing the proper preventive management strategies for safe manufacture and use.

## Author Contributions

All authors listed have made a substantial, direct and intellectual contribution to the work, and approved it for publication.

### Conflict of Interest Statement

The authors declare that the research was conducted in the absence of any commercial or financial relationships that could be construed as a potential conflict of interest.

## References

[B1] Amaranatha R. D. Ma R. Choi M. Y. Kim T. K. (2015). Reduced graphene oxide wrapped ZnS-Ag_2_S ternary composites synthesized via hydrothermal method: applications in photocatalyst degradation of organic pollutants. Appl. Surf. Sci. 324, 725–735. 10.1016/j.apsusc.2014.11.026

[B2] Anjum N. A. Singh N. Singh M. K. Sayeed I. Duarte A. C. Pereira E.. (2014). Single-bilayer graphene oxide sheet impacts and underlying potential mechanism assessment in germinating faba bean (*Vicia faba* L.). Sci. Total Environ. 472, 834–841. 10.1016/j.scitotenv.2013.11.018 24342089

[B3] Anjum N. A. Singh N. Singh M. K. Shah Z. A. Duarte A. C. Pereira E. (2013). Single-bilayer graphene oxide sheet tolerance and glutathione redox system significance assessment in faba bean (*Vicia faba* L.). J. Nanopart. Res. 15, 1–12. 10.1007/s11051-013-1770-7

[B4] Arvidsson R. Molander S. Sandén B. A. (2013). Review of potential environmental and health risks of the nanomaterial graphene. Hum. Ecol. Risk Assess. 19, 873–887. 10.1080/10807039.2012.702039

[B5] Asli S. Neumann P. M. (2009). Colloidal suspensions of clay or titanium dioxide nanoparticles can inhibit leaf growth and transpiration via physical effects on root water transport. Plant Cell Environ. 32, 577–584. 10.1111/j.1365-3040.2009.01952.x 19210640

[B6] Begurn P. Ikhtiari R. Fugetsu B. (2011). Graphene phytotoxicity in the seedling stage of cabbage, tomato, red spinach, and lettuce. Carbon 49, 3907–3919. 10.1016/j.carbon.2011.05.029

[B7] Cañas J. E. Long M. Nations S. Vadan R. Dai L. Luo M.. (2008). Effects of functionalized and nonfunctionalized single-walled carbon nanotubes on root elongation of select crop species. Environ. Toxicol. Chem. 27, 1922–1931. 10.1897/08-117.1 19086209

[B8] Chen J. Yang L. Li S. Ding W. (2018a). Various physiological response to graphene oxide and amine-functionalized graphene oxide in wheat (*Triticum aestivum*). Molecules 23 1104. 10.3390/molecules23051104 29735929 PMC6100068

[B9] Chen L. Wang C. Li H. Qu X. Yang S. Chang X. (2017). Bioaccumulation and toxicity of ^13^C-skeleton labeled graphene oxide in wheat. Environ. Sci. Technol. 51, 10146–10153. 10.1021/acs.est.7b00822 28771335

[B10] Chen L. Wang C. Yang S. Guan X. Zhang Q. Shi M. (2019). Chemical reduction of graphene enhances *in vivo* translocation and photosynthetic inhibition in pea plants. Environ. Sci-Nano. 6, 1077–1088. 10.1039/C8EN01426D

[B11] Chen M. Zhou S. Zhu Y. Sun Y. Zeng G. Yang C.. (2018b). Toxicity of carbon nanomaterials to plants, animals and microbes: recent progress from 2015-present. Chemosphere 206, 255–264. 10.1016/j.chemosphere.2018.05.020 29753288

[B12] Chen Z. Umar A. Wang S. Wang Y. Tian T. Shang Y.. (2015). Supramolecular fabrication of multilevel graphene-based gas sensors with high NO_2_ sensibility. Nanoscale 7, 10259–10266. 10.1039/C5NR01770J 25990644

[B13] Cheng F. Liu Y. Lu G. Zhang X. Xie L. Yuan C.. (2016). Graphene oxide modulates root growth of *Brassica napus* L. and regulates ABA and IAA concentration. J. Plant Physiol. 193, 57–63. 10.1016/j.jplph.2016.02.011 26945480

[B14] Chowdhury S. Balasubramanian R. (2014). Recent advances in the use of graphene-family nanoadsorbents for removal of toxic pollutants from wastewater. Adv. Colloid Interface 204, 35–56. 10.1016/j.cis.2013.12.005 24412086

[B15] De La Torre-Roche R. Hawthorne J. Deng Y. Xing B. Cai W. Newman L. A.. (2012). Fullerene-enhanced accumulation of p,p′-DDE in agricultural crop species. Environ. Sci. Technol. 46, 9315–9323. 10.1021/es301982w 22856886

[B16] Dreyer D. R. Park S. Bielawski C. W. Ruoff R. S. (2010). The chemistry of graphene oxide. Chem. Soc. Rev. 39, 228–240. 10.1039/B917103G 20023850

[B17] Gilbert N. (2009). Nanoparticle safety in doubt. Nature 460:937. 10.1038/460937a 19693048

[B18] Gobbi M. Bonacchi S. Lian J. X. Liu Y. Wang X. Stoeckel M.. (2017). Periodic potentials in hybrid van der Waals heterostructures formed by supramolecular lattices on graphene. Nat. Commun. 8:14767. 10.1038/ncomms14767 28322229 PMC5364416

[B19] Hao Y. Ma C. Zhang Z. Song Y. Cao W. Guo J.. (2018). Carbon nanomaterials alter plant physiology and soil bacterial community composition in a rice-soil-bacterial ecosystem. Environ. Pollut. 232, 123–136. 10.1016/j.envpol.2017.09.024 28947315

[B20] He Y. Hu R. Zhong Y. Zhao X. Chen Q. Zhu H. (2018). Graphene oxide as a water transporter promoting germination of plants in soil. Nano Res. 11, 1928–1937. 10.1007/s12274-017-1810-1

[B21] Hu X. Kang J. Lu K. Zhou R. Mu L. Zhou Q. (2014a). Graphene oxide amplifies the phytotoxicity of arsenic in wheat. Sci. Rep. 4:6122. 10.1038/srep06122 25134726 PMC4137339

[B22] Hu X. Lu K. Mu L. Kang J. Zhou Q. (2014b). Interactions between graphene oxide and plant cells: Regulation of cell morphology, uptake, organelle damage, oxidative effects and metabolic disorders. Carbon 80, 665–676. 10.1016/j.carbon.2014.09.010

[B23] Hu X. Mu L. Kang J. Lu K. Zhou R. Zhou Q. (2014c). Humic acid acts as a natural antidote of graphene by regulating nanomaterial translocation and metabolic fluxes *in vivo*. Environ. Sci. Technol. 48, 6919–6927. 10.1021/es5012548 24857237

[B24] Hu X. Zhou Q. (2014). Novel hydrated graphene ribbon unexpectedly promotes aged seed germination and root differentiation. Sci. Rep. 4:3782. 10.1038/srep03782 24445438 PMC3896910

[B25] Huang C. Xia T. Niu J. Yang Y. Lin S. Wang X. (2018). Transformation of ^14^C-labeled graphene to ^14^CO_2_ in the shoots of a rice plant. Angew. Chem. Int. Edit. 57, 9759–9763. 10.1002/anie.201805099 29928789

[B26] Kotchey G. P. Allen B. L. Vedala H. Yanamala N. Kapralov A. A. Tyurina Y. Y.. (2011). The enzymatic oxidation of graphene oxide. ACS Nano 5, 2098–2108. 10.1021/nn103265h 21344859 PMC3062704

[B27] Lee J. H. Han J. H. Kim J. H. Kim B. Bello D. Kim J. K.. (2016). Exposure monitoring of graphene nanoplatelets manufacturing workplaces. Inhal. Toxicol. 28, 281–291. 10.3109/08958378.2016.1163442 27055369

[B28] Lingamdinne L. P. Koduru J. R. Roh H. Choi Y. Chang Y. Yang J. (2016). Adsorption removal of Co(II) from waste-water using graphene oxide. Hydrometallurgy 165, 90–96. 10.1016/j.hydromet.2015.10.021

[B29] Liu S. Wei H. Li Z. Li S. Yan H. He Y.. (2015). Effects of graphene on germination and seedling morphology in rice. J. Nanosci. Nanotechno. 15, 2695–2701. 10.1166/jnn.2015.9254 26353483

[B30] Miralles P. Church T. L. Harris A. T. (2012). Toxicity, uptake, and translocation of engineered nanomaterials in vascular plants. Environ. Sci. Technol. 46, 9224–9239. 10.1021/es202995d 22892035

[B31] Naasz S. Altenburger R. Kuehnel D. (2018). Environmental mixtures of nanomaterials and chemicals: The Trojan-horse phenomenon and its relevance for ecotoxicity. Sci. Total Environ. 635, 1170–1181. 10.1016/j.scitotenv.2018.04.180 29710572

[B32] Nair R. Mohamed M. S. Gao W. Maekawa T. Yoshida Y. Ajayan P. M.. (2012). Effect of carbon nanomaterials on the germination and growth of rice plants. J. Nanosci. Nanotechno. 12, 2212–2220. 10.1166/jnn.2012.5775 22755040

[B33] Nel A. Xia T. Mädler L. Li N. (2006). Toxic potential of materials at the nanolevel. Science 311, 622. 10.1126/science.1114397 16456071

[B34] Ou L. Song B. Liang H. Liu J. Feng X. Deng B.. (2016). Toxicity of graphene-family nanoparticles: a general review of the origins and mechanisms. Part. Fibre Toxicol. 13:57. 10.1186/s12989-016-0168-y 27799056 PMC5088662

[B35] Pirveysian M. Ghiaci M. (2018). Synthesis and characterization of sulfur functionalized graphene oxide nanosheets as efficient sorbent for removal of Pb^2+^, Cd^2+^, Ni^2+^ and Zn^2+^ ions from aqueous solution: A combined thermodynamic and kinetic studies. Appl. Surf. Sci. 428, 98–109. 10.1016/j.apsusc.2017.09.105

[B36] Sanchez V. C. Jachak A. Hurt R. H. Kane A. B. (2012). Biological interactions of graphene-family nanomaterials: an interdisciplinary review. Chem. Res. Toxicol. 25, 15–34. 10.1021/tx200339h 21954945 PMC3259226

[B37] Suárez-Iglesias O. Collado S. Oulego P. Díaz M. (2017). Graphene-family nanomaterials in wastewater treatment plants. Chem. Eng. J. 313, 121–135. 10.1016/j.cej.2016.12.022

[B38] Sun M. Jiang F. Cen B. Wen J. Zhou Y. Wu Z. (2018). Respiratory burst oxidase homologue-dependent H_2_O_2_ and chloroplast H_2_O_2_ are essential for the maintenance of acquired thermotolerance during recovery after acclimation. Plant Cell Environ. 41, 2373–2389. 10.1111/pce.13351 29851102

[B39] Tan P. Sun J. Hu Y. Fang Z. Bi Q. Chen Y. (2015). Adsorption of Cu^2+^, Cd^2+^ and Ni^2+^ from aqueous single metal solutions on graphene oxide membranes. J. Hazard. Mater. 297, 251–260. 10.1016/j.jhazmat.2015.04.068 25978188

[B40] Thomas K. Sayre P. (2005). Research strategies for safety evaluation of nanomaterials, Part I: evaluating the human health implications of exposure to nanoscale materials. Toxicol. Sci. 87, 316–321. 10.1093/toxsci/kfi270 16049265

[B41] Wang X. Xie H. Wang Z. He K. Jing D. (2019). Graphene oxide as a multifunctional synergist of insecticides against lepidopteran insect. Environ. Sci. Nano 6, 75–84. 10.1039/C8EN00902C

[B42] Wiesner M. R. Lowry G. V. Jones K. L. Hochella J. M. F. Di Giulio R. T. Casman E.. (2009). Decreasing uncertainties in assessing environmental exposure, risk, and ecological implications of nanomaterials. Environ. Sci. Technol. 43, 6458–6462. 10.1021/es803621k 19764202

[B43] Xing W. Lalwani G. Rusakova I. Sitharaman B. (2014). Degradation of graphene by hydrogen peroxide. Part. Part. Syst. Char. 31, 745–750. 10.1002/ppsc.201300318

[B44] Xu J. Cao Z. Zhang Y. Yuan Z. Lou Z. Xu X.. (2018). A review of functionalized carbon nanotubes and graphene for heavy metal adsorption from water: Preparation, application, and mechanism. Chemosphere 195, 351–364. 10.1016/j.chemosphere.2017.12.061 29272803

[B45] Yan J. Hu C. Chen K. Lin Q. (2019). Release of graphene from graphene-polyethylene composite films into food simulants. Food Packaging Shelf 20:100310 10.1016/j.fpsl.2019.100310

[B46] Yang K. Xing B. (2007). Desorption of polycyclic aromatic hydrocarbons from carbon nanomaterials in water. Environ. Pollut. 145, 529–537. 10.1016/j.envpol.2006.04.020 16777283

[B47] Yang X. P. Zhao F. (2013). A review of uptake, translocation and phytotoxicity of engineered nanoparticles in plants. Environ. Sci. 34, 4495–4502 10.13227/j.hjkx.2013.11.005 24455965

[B48] Yin L. Wang Z. Wang S. Xu W. Bao H. (2018). Effects of graphene oxide and/or Cd^2+^ on seed germination, seedling growth, and uptake to Cd^2+^ in solution culture. Water Air Soil Poll. 229, 151 10.1007/s11270-018-3809-y

[B49] Zhang C. Zhang R. Z. Ma Y. Q. Guan W. B. Wu X. L. Liu X. (2015a). Preparation of cellulose/graphene composite and its applications for triazine pesticides adsorption from water. ACS Sustain. Chem. Eng. 3, 396–405. 10.1021/sc500738k

[B50] Zhang M. Gao B. Chen J. Li Y. (2015b). Effects of graphene on seed germination and seedling growth. J. Nanopart. Res. 17:78 10.1007/s11051-015-2885-9

[B51] Zhang P. Zhang R. Fang X. Song T. Cai X. Liu H.. (2016). Toxic effects of graphene on the growth and nutritional levels of wheat (*Triticum aestivum* L.): short- and long-term exposure studies. J. Hazard. Mater. 317, 543–551. 10.1016/j.jhazmat.2016.06.019 27343870

[B52] Zhang Z. Hu J. Liu S. Zhang C. Liu X. Ye C. (2018). Effect of nano-SiO_2_ on the toxicity of Hg^2+^ to *Skeletonema costatum*. Environ. Chem. 37, 661–669 10.7524/j.issn.0254-6108.2017081506

[B53] Zhao J. Cao X. Wang Z. Dai Y. Xing B. (2017). Mechanistic understanding toward the toxicity of graphene-family materials to freshwater algae. Water Res. 111, 18–27. 10.1016/j.watres.2016.12.037 28040538

[B54] Zhao J. Wang Z. White J. C. Xing B. (2014). Graphene in the aquatic environment: Adsorption, dispersion, toxicity and transformation. Environ. Sci. Technol. 48, 9995–10009. 10.1021/es5022679 25122195

[B55] Zhao S. Wang Q. Zhao Y. Rui Q. Wang D. (2015). Toxicity and translocation of graphene oxide in *Arabidopsis thaliana*. Environ. Toxicol. Phar. 39, 145–156. 10.1016/j.etap.2014.11.014 25499792

[B56] Zhou J. Chen M. Diao G. (2013). Calix[4,6,8]arenesulfonates functionalized reduced graphene oxide with high supramolecular recognition capability: Fabrication and application for enhanced host-guest electrochemical recognition. ACS Appl. Mater. Inter. 5, 828–836. 10.1021/am302289v 23311992

